# Research Progress of Biosensors in the Detection of Pesticide Residues and Heavy Metals in Tea Leaves

**DOI:** 10.3390/bios15120778

**Published:** 2025-11-26

**Authors:** Pin Li, Miaopeng Chen, Tianle Yao, Long Wu, Shanran Wang, Yu Han, Ying Song, Jia Yin

**Affiliations:** 1 Qinghai Provincial Laboratory for Intelligent Computing and Application, School of Energy and Electrical Engineering, Qinghai University, Xining 810016, Chinachenmiaopeng_0727@163.com (M.C.); 2 Hubei Provincial Institute for Food Supervision and Test, Wuhan 430075, China; hbyinjia@163.com; 3 Hubei Key Laboratory of Resource Utilization and Quality Control of Characteristic Crops, College of Life Science and Technology, Hubei Engineering University, Xiaogan 432000, China; ytl20031225@163.com; 4 Key Laboratory of Tropical Fruits and Vegetables Quality and Safety for State Market Regulation, School of Food Science and Engineering, Hainan University, Haikou 570228, China; wulong@hainanu.edu.cn; 5 School of Aeronautic Science and Engineering, Beihang University, Beijing 100191, China; wang_shanran@buaa.edu.cn

**Keywords:** food safety, pollution, tea, biosensing technology, analytical method

## Abstract

Tea, a worldwide prevalent beverage, is continually contaminated by pesticide residues and heavy metals, presenting considerable health concerns to consumers. Nonetheless, effective monitoring is limited by conventional detection techniques—such as gas chromatography (GC) and inductively coupled plasma mass spectrometry (ICP-MS)—which, despite their high precision, necessitate intricate pretreatment, incur substantial operational expenses, and are inadequate for swift on-site analysis. Biosensors have emerged as a viable option, addressing this gap with their exceptional sensitivity, rapid response, and ease of operation.This review rigorously evaluates recent advancements in biosensing technologies for the detection of pesticide residues and heavy metals in tea, emphasizing the mechanisms, analytical performance, and practical applicability of prominent platforms such as fluorescence, surface-enhanced Raman scattering (SERS), surface plasmon resonance (SPR), colorimetric, and electrochemical biosensors. Electrochemical and fluorescent biosensors provide the highest promise for portable, on-site use owing to their enhanced sensitivity, cost-effectiveness, and flexibility to intricate tea matrices. The paper further emphasizes upcoming techniques such multi-component detection, microfluidic integration, and AI-enhanced data processing. Biosensors provide significant potential to revolutionize tea safety monitoring, with future advancements dependent on the synergistic incorporation of sophisticated nanomaterials, intelligent microdevices, and real-time analytics across the whole “tea garden-to-cup” supply chain.

## 1. Introduction

Tea, a worldwide prevalent non-alcoholic beverage, has cultural importance across many areas and historical circumstances. It is preferred by consumers globally for its health advantages, including antioxidant effects and metabolic control, which are ascribed to its abundant bioactive constituents including tea polyphenols and catechins [1]. Industry statistics indicate that global yearly tea consumption has surpassed 6 million tons [2]. China, as the foremost producer and consumer of tea, holds an indispensable strategic role in fostering agricultural economic development, enhancing rural employment, and facilitating rural regeneration [3,4].

**Figure 1:**
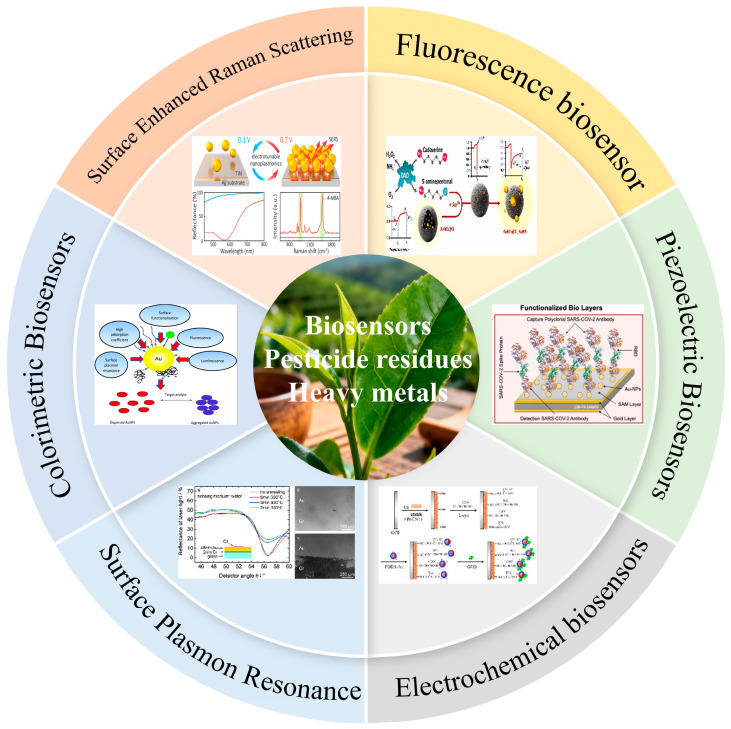
Graphical visualization of the framework for developing technologies for tea safety and detection. All figures in this diagram illustrate the framework and working principles of various biosensing technologies for tea safety monitoring.

However, as tea production increases and worldwide consumption rises (Figure 1), the concerns of quality and safety in tea have become more important [5]. Figure 1 presents a schematic representation of the structure and operational principles of several biosensing technologies used for monitoring tea safety. Pesticide residues and heavy metal pollution have become significant barriers to improving tea quality and provide health hazards to consumers. The complete tea supply chain, encompassing cultivation to processing, poses a risk of contaminant introduction, with pesticide residues predominantly stemming from the application of organophosphorus compounds, pyrethroids, neonicotinoids, and various other chemical agents in agricultural pest control. Heavy metals, such as lead, cadmium, and mercury, permeate tea plants via industrial emissions, soil buildup, and the use of polluted agricultural inputs, including inferior fertilizers. Moreover, microbiological contamination, shown by Escherichia coli, mycotoxin contamination, including aflatoxin, and fluoride presence in some teas, such as brick tea, pose possible hazards to tea safety. These pollutants cause the deterioration of tea aroma, modification of soup color, and reduction in sensory quality and commercial value, while also accumulating in the human body through extended dietary intake, resulting in neurotoxicity, hepatic and renal damage, and an increased risk of chronic diseases and cancer [6,7].

A rigorous standardization system for tea safety control has been implemented both domestically and internationally. The existing national food safety standards in China—the National Food Safety Standard for Maximum residual Limits of Pesticides in Foods (GB 2763-2021) [8] and the National Food Safety Standard for Tea (GB 31608-2023) [9]—specify the residual limits for several pesticides and heavy metals [6]. At the international level, Regulation (EC) No 396/2005 of the European Union and the Japanese Positive List System have established stringent residual thresholds for imported tea. Nonetheless, the frequent alerts concerning Chinese tea exports to the EU (2020–2023) related to excessive pesticide residues underscored the deficiencies in the existing tea quality monitoring framework and detection methodologies [10,11].

The current methods for detecting tea pollutants primarily rely on chromatography (GC, HPLC), mass spectrometry (ICP-MS), and their integrated techniques [12]. Despite their high accuracy and low detection limits, these methods necessitate intricate sample pretreatment processes, including solid-phase extraction and microwave digestion, as well as substantial equipment investments, with the cost of a single ICP-MS exceeding one million RMB. Additionally, they entail prolonged detection cycles and pose challenges for on-site rapid screening, significantly constraining their applicability in the preliminary screening of large-scale samples and real-time monitoring of tea garden fields. Consequently, the advancement of efficient, sensitive, and portable technology for detecting tea pollutants has emerged as a focal point of study within the domain of food science and analysis.

The emergence of biosensor technology offers a novel answer to this need. This technology integrates highly specific biological recognition elements, including enzymes, monoclonal antibodies, and nucleic acid aptamers, with sensitive signal conversion systems utilizing light, electricity, and sound. It exhibits high sensitivity with detection limits ranging from nM to pM, strong specificity resistant to interference from tea polyphenols, alkaloids, and other matrix components, rapid response times of 5–30 min, and potential for portability, positioning it as a pivotal alternative to conventional detection methods [13,14]. This system allows for real-time quantitative detection of trace pesticide residues and heavy metals in tea, while enabling simultaneous multi-component analysis through a multi-channel architecture, thus providing advanced technical support for on-site monitoring and source management of tea quality and safety. Biosensors are progressing towards improved sensitivity, intelligence, and integration, particularly via the inclusion and use of nanomaterials such as metal–organic frameworks (MOFs) and gold nanoparticles, as well as microfluidic chips and artificial intelligence technologies. A comprehensive tea safety monitoring network encompassing the full “tea garden-to-cup” chain is expected to be built in the future to facilitate the high-quality progress of the global tea industry.

## 2. Types and Sources of Pesticide Residues and Heavy Metals in Tea

This study thoroughly investigates the two primary safety indicators of pesticide residues and heavy metals in tea by species classification and source analysis. The examination of pesticide residues, in conjunction with alterations in chemical structures, toxicity risk thresholds, and pollution transmission mechanisms, resulted in the discovery of primary types of pesticide residues, including pyrethroids [15] and organophosphorus compounds [16]. A thorough analysis of their source characteristics revealed both primary pollution resulting from the direct application of pesticides in tea garden cultivation and secondary pollution arising from environmental migration processes such as atmospheric deposition and water flow [17], as well as cross-contamination during processing stages and compliance risks associated with differing national and regional trade standards. In the realm of heavy metal research, we concentrate on high-risk metals such as lead and cadmium, categorizing their many sources based on toxicity, bioaccumulation, and environmental migration potential. Geological background circumstances, including soil parent material composition and regional hydrological dynamics [18], are the primary determinants of heavy metal background levels. Heavy metal contamination in tea results from anthropogenic activities, industrial waste discharge, overuse of agricultural inputs, and metal leaching from processing equipment. The aforementioned study provides a vital theoretical basis for accurate identification, targeted prevention, and control of tea contaminants.

### 2.1. Classification and Properties of Tea Pesticide Residues

Various characteristics may be used to systematically categorize the kinds of pesticide residues present in tea (Table 1), according to the established categorization standards of the Food and Agriculture Organization (FAO) and the World Health Organization (WHO). According to the FAO/WHO categorization system, which first categorizes pesticides by their target and then subdivides them by chemical structure, these residues may be classified into four separate groups depending on their chemical composition and mechanism of action [19]: One is pyrethroids [15]. This chemical functions as an insecticide by disrupting the insect nerve conduction system and exhibits the maximum detection frequency in white tea. The detection rate of bifenthrin is 26.4%, while that of fenpropathrin is 21.7%. However, fenvalerate [20] has been classified as a prohibited pesticide for tea cultivation because of its environmental persistence. The second category include organophosphorus insecticides and carbamates [16]. Omethoate and carbofuran exemplify compounds that have insecticidal effects by reducing the action of insect acetylcholinesterase. Their considerable toxicity has severely restricted their field of applicability. The third is nicotine and heterocyclic pesticides [21], which target on the insect nicotinic acetylcholine receptor. Imidacloprid and dinotefuran had respective detection rates of 15.1% and 6.67%. The fourth is fungicides and herbicides, which are frequently used in conjunction with tolfenpyrad to improve the control effect in practical situations [22]. Carbendazim (detection rate 15.1%) is mostly employed to manage tea cake disease, while glyphosate (detection rate 9.4%) is utilized for weed management. Due to their water-soluble properties, they readily infiltrate the soil via the irrigation system and are assimilated by the roots of tea plants.

Tea pesticide residues are classified into three categories based on toxicity and usage regulations: banned, restricted, and permitted [35,36]. Pesticides, including fenvalerate and dicofol, are completely forbidden throughout the tea production process. The use of restricted pesticides, such as glyphosate, must conform rigorously to designated dosage limits and safety intervals. The approved pesticides include bifenthrin and imidacloprid, among others, and their residues must adhere to the National Food Safety Standard for Maximum Residue Limits of Pesticides in Foods (GB 2763-2021) [6,8].

Pesticide residues in tea may be categorized into two groups according to the mode of pollutant transmission: direct pollution and indirect pollution. Direct contamination mostly arises from the use of pesticides, including pyrethroids, on tea leaves in the production of tea plantations. Indirect contamination refers to the transmission of pesticides to tea plants via environmental channels, such as soil adsorption and water movement, often shown by glyphosate and DDT (dichlorodiphenyltrichloroethane).

### 2.2. Primary Sources and Contamination Pathways

**Figure 2:**
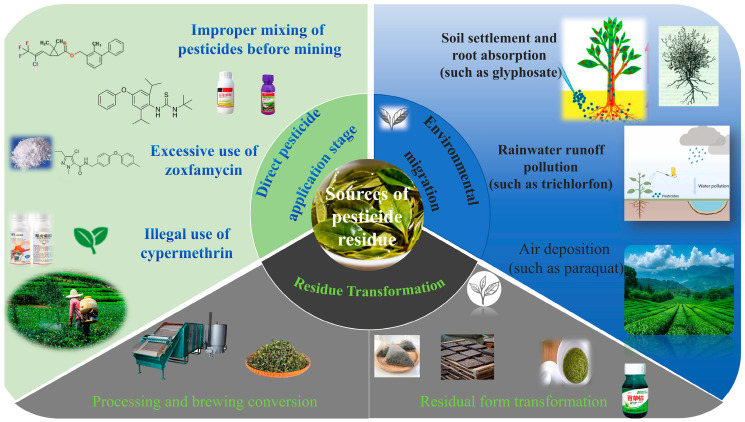
Illustrations of pathways of pesticide contamination in tea: soil uptake, mis-timed spraying, runoff, illegal use, airborne deposition, and solubility-driven residue transfer.

The sources of pesticide residues in tea shown significant diversity (Figure 2). The widespread occurrence of non-standard pesticide application procedures is a primary cause leading to the violation of pesticide residue requirements. In contemporary tea garden management, some tea farmers often amalgamate bifenthrin [23] with diafenthiuron to attain improved control effectiveness, perhaps leading to a synergistic residual effect. The unlawful use of pesticides shortly before tea harvesting has resulted in pesticide residues in fresh leaves significantly beyond safety guidelines. The maximum authorized dosage of tolfenpyrad [37] is 525 g/hm^2^. If tea farmers exceed recommended dosages or fail to adhere to the safety interval (for instance, harvesting within 7 days post-application), the residue of tolfenpyrad in dried tea products is likely to surpass permissible limits. Furthermore, while fenvalerate is designated as a banned pesticide for tea growing, several tea farmers persist in its unlawful use owing to its broad insecticidal spectrum and cost-effectiveness, hence increasing the likelihood of pesticide residues in tea.

Environmental migration constitutes a significant source of pesticide residues in tea [17]. During pesticide application procedures, approximately 60% to 70% of the pesticides are deposited onto the soil surface. For instance, glyphosate [38] has a half-life in soil of up to 180 days and can be absorbed by the roots of tea plants, then translocating to the buds and leaves due to its internal absorption properties. Pesticides used in the agricultural areas next to the tea garden may infiltrate the irrigation water supply via rainfall runoff, resulting in the detection of pesticide varieties not directly applied inside the tea garden, such as dicofol. Pesticides used on adjacent agricultural fields, such as paraquat, may contaminate tea gardens by atmospheric volatilization, diffusion, and aerial deposition [39], further complicating the issue of tea pesticide residues.

The processing connections significantly influence the final pesticide residue in tea. During the tea drying process, certain pesticides will become concentrated as a result of water evaporation. The drying factor of dinotefuran [40] reaches 258%, leading to a considerably greater residue in dried tea compared to fresh leaves. Regarding pesticide solubility, the leaching rate of water-soluble pesticides [41], such as dinotefuran, during the brewing process can attain 93.5%, whereas the leaching rate of fat-soluble pesticides [42], such as tolfenpyrad, in tea soup is minimal and nearly insoluble. This suggests that both processing techniques and brewing practices influence the actual consumption of pesticide residues by consumers.

The variation in pesticide residue standards in global commerce highlights the necessity for rigorous oversight of pesticide residues in tea. The EU and several foreign markets have implemented stringent residual limits for certain pesticides, such as dinotefuran and tolfenpyrad, and have adopted a “zero tolerance” approach for unapproved pesticides. From 2020 to 2025, the European Union filed 23 notices over China’s tea exports owing to excessive pesticide residues, leading to direct economic losses above 120 million Chinese yuan. This environmental trade barrier has compelled China’s tea sector to expedite the refinement of the agrochemical composition in tea gardens and actively advocate for the use of new pesticides characterized by high efficacy, low toxicity, and minimal residue [43], thereby augmenting the worldwide competitiveness of tea products. The multi-dimensional analysis of tea pesticide residue sources elucidates the complexity of the pesticide residue issue and offers a crucial scientific foundation for developing tailored preventative and control techniques.

### 2.3. Heavy Metal Species in Tea

The classification of heavy metals in tea can be systematically categorized across various dimensions (Table 2), with sources stemming from both natural processes and anthropogenic activities [44]. Heavy metals in tea can be classified into three categories based on their chemical properties and toxicity levels. The first category comprises highly toxic heavy metals, which includes lead (Pb), cadmium (Cd), mercury (Hg), hexavalent chromium (Cr^6+^), and metalloid arsenic (As). Heavy metals exhibit significant biological toxicity. Lead can induce anemia by inhibiting heme synthase activity, while cadmium can impair renal tubular function [45,46]. Consequently, their residues are rigorously regulated by applicable EU standards, with the maximum residue limit for lead set at 5.0 mg/kg [47]. Moderately toxic heavy metals include copper (Cu), zinc (Zn), and nickel (Ni). While essential trace elements are crucial for the growth of tea plants, excessive accumulation can disrupt the activity of the body’s enzyme system [48]. When the copper content in dry tea surpasses 30 mg/kg, it may lead to gastrointestinal discomfort in humans [49,50]. The third category pertains to heavy metals that necessitate attention, as their toxicity is closely associated with their various chemical forms. The toxicity of Cr^6+^ is 100 times greater than that of Cr^3+^, and organic mercury exhibits significantly higher toxicity to the nervous system compared to inorganic mercury [51].

From the standpoint of environmental migration characteristics, heavy metals in tea can be classified into two categories: those exhibiting high mobility and those demonstrating low mobility. Cationic heavy metals, including cadmium (Cd) and zinc (Zn), readily form complexes with soil organic matter and are actively taken up by tea roots via ion exchange. The bio-concentration factor (BCF) varies between 0.1 and 0.5, while the transfer factor (TF) is ranked as follows: cadmium (0.3–0.6) > zinc (0.1–0.3) > lead (0.01–0.05), indicative of high-mobility heavy metals [51]. Heavy metals like lead (Pb) and chromium (Cr) readily form hydroxide precipitates in soil, exhibiting limited migration capacity. Lateral diffusion is primarily accomplished through surface runoff via soil colloid adsorption [52].

Based on the source of pollution, heavy metal contamination in tea can be categorized into primary and secondary pollution. The principal source of pollution mostly originates from the natural release processes of the crust of the earth [53]. The lead (Pb) concentration in soil derived from granite parent material ranges from 50 to 80 mg/kg, while the chromium (Cr) concentration in soil from basalt parent material varies from 50 to 150 mg/kg. Human activities [54] are the primary source of secondary pollution, which significantly contributes to the elevated levels of heavy metals in tea.

### 2.4. Source Analysis of Heavy Metals in Tea

**Figure 3:**
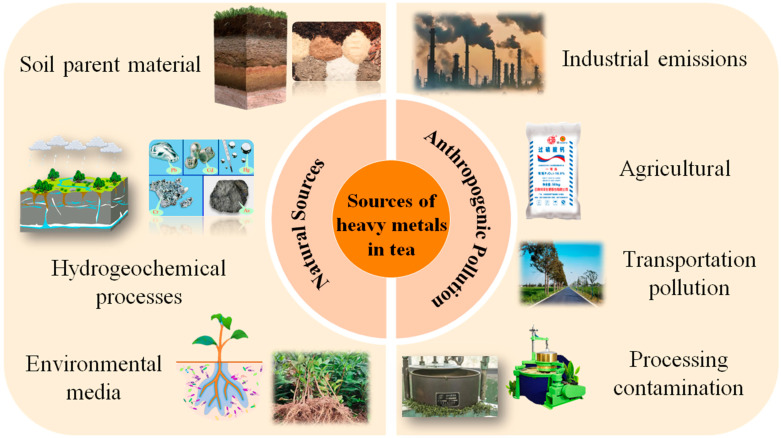
Pathways of heavy-metal entry into tea: parent rock, airfall dust, fertilizer-derived cadmium, traffic emissions, soil–water transport, root mobilization, and processing wear.

The sources of heavy metals in tea may be systematically classified into two categories: natural geological background and anthropogenic pollution, both of which affect the accumulation of heavy metals in tea via different mechanisms (Figure 3).

#### 2.4.1. Source of Natural Geological Background

The natural geological backdrop is the primary determinant of the baseline levels of heavy metals in tea [18]. The geochemical properties of soil parent materials directly limit the initial levels of heavy metals. Soil originating from shale readily accumulates cadmium, with a baseline concentration of 0.3–0.8 mg/kg. In contrast, soil derived from basalt parent material has elevated chromium contents, ranging from 50 to 150 mg/kg. The dynamic regional geological structure, extensive rock weathering, and prominent geochemical cycling of elements contribute to elevated background levels of heavy metals in soil in the southwestern tea area of China compared to the eastern tea region [55].

Hydrogeochemistry significantly influences the bioavailability of heavy metals [56]. In acidic soil conditions (pH < 5.5), hydrogen ions can facilitate the desorption of heavy metal ions from the soil colloid surface, resulting in lead availability that is 3–4 times greater than in neutral soil (pH 6.5). Simultaneously, precipitation leaching and groundwater penetration will expedite the translocation of soluble heavy metals to the root distribution zone of tea plants [57].

These mechanisms provide the basis for the exchange of heavy metals throughout the ecosystem of the tea area. In the soil–plant system, heavy metals are assimilated by tea plants via their roots, with the transfer coefficient (TF) ranked as follows: Cd (0.3–0.6) > Zn (0.1–0.3) > Pb (0.01–0.05). Organic acids, including citric acid in root exudates, can enhance the solubility and bioavailability of lead in soil via complexation. At the air-plant interface, lead-containing particles smaller than 2.5 μm can infiltrate the plant via leaf stomata and accumulate in the sponge tissue, leading to a lead concentration on the leaf surface that is 2–3 times greater than that on the underside of the leaves. The efficacy of migration is closely associated with the biological characteristics of tea leaves, including the density of surface villi and stomatal conductance [58].

#### 2.4.2. Source of Pollutants

Human-induced pollution is the principal cause of increased heavy metal concentrations in tea, mostly stemming from industrial operations, farming practices, transportation, and processing.

Industrial activities release heavy metals that contribute to regional pollution via multi-media diffusion [59]. Lead-containing dust from iron and steel plants and smelters is deposited in tea gardens via dry atmospheric deposition, resulting in lead concentrations in tea leaves within 5 km of the pollution source that are 2–3 times higher than background levels. Fine particulate matter (PM2.5) can facilitate the direct absorption of heavy metals into the tea plant via leaf adsorption. The presence of chromium in electroplating effluent may cause soil contamination via the irrigation system, possibly leading to tea chromium levels exceeding 30%.

The prolonged excessive application of agricultural inputs markedly increases the accumulation of heavy metals in soil [60]. The concentration of cadmium in calcium super-phosphate may reach 1–5 mg/kg, with long-term application resulting in an annual soil cadmium accumulation rate of 0.02 mg/kg, which can be absorbed by tea plants through their root systems. Additionally, the historical application of arsenic-containing fungicides, such as thiram, has increased arsenic levels in certain tea garden soils to 15–20 mg/kg, with a half-life of arsenic in the soil ranging from 100 to 200 years, thereby presenting a long-term potential risk to tea safety.

Transportation-induced heavy metal pollution exhibits notable spatial gradient characteristics [61]. The lead content in tea at 20 m from both sides of the highway (1.25 mg/kg) was 1.8 times greater than that at 80 m. The zinc released from tire wear is deposited into the soil via atmospheric processes, resulting in a zinc concentration in the soil near traffic trunk lines that is 40% greater than that found in control areas.

The possibility of heavy metal contamination in tea manufacturing is considerable and requires consideration. The deterioration of copper rolling machines may produce copper chips, thereby elevating the copper concentration in dry tea over the acceptable threshold of 30 mg/kg set by the National Food Safety Standard for Maximum Levels of Contaminants in Foods (GB 2762-2022) [30] by more than 15%. Conversely, using stainless steel equipment may reduce the likelihood of copper contamination by 60% [62]. If the lead content in talcum powder used for frying surpasses the standard (>10 mg/kg), the lead content in tea will rise by 0.5–1.0 mg/kg. The degree of pollution exhibits a positive correlation with the contact area of excipients and tea, as well as the processing temperature.

## 3. The Harm and Influence of Pesticide Residues and Heavy Metals in Tea Leaves

**Figure 4:**
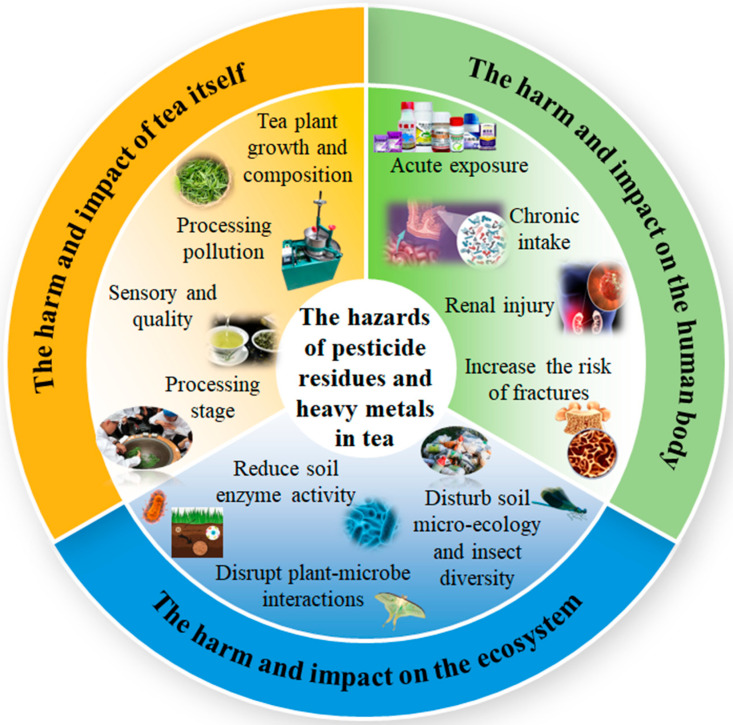
Chronic pollution- and processing-stage contaminant-induced impairment of tea quality via soil micro-ecology and plant–microbe disruption, with associated consumer renal injury and fracture risk elevation.

This section systematically analyzes the dangers and impacts of pesticide residues and heavy metals in tea from three essential perspectives: “tea, human health, ecosystem” (Figure 4): Firstly, it clarifies the direct disruption and deterioration of physiological metabolism, sensory characteristics, and processing properties of tea. Additionally, assess the toxicity hazards to the human body either from acute exposure or chronic use [63] including potential nerve damage, organ damage, and carcinogenic risk. Ultimately, it elucidates the functional imbalance and chain hazards resulting from multimedia migration and biomagnification effects on soil [64], as well as aquatic and terrestrial ecosystems. This research comprehensively delineates the total effects of these two pollutant kinds on the tea industry supply chain and its adjacent environment.

### 3.1. Hazards and Effects on Tea

Pesticide residues interfere with the physiological metabolism and quality development of tea via many processes. Organochlorine pesticides, such as DDT, build in the lipid constituents of tea owing to their lipid solubility, leading to unpleasant aromas in tea infusion and masking the natural fragrance of tea leaves [65]. Additionally, the water solubility of glyphosate can inhibit the activity of tea polyphenol oxidase, leading to a dull color in green tea soup [66]. Neonicotinoid pesticides, including imidacloprid, inhibit the cytochrome P450 enzyme system in tea plants, which impedes caffeine synthesis and results in a 15–20% reduction in caffeine content in dry tea. The drying concentration effect during processing results in a 2.58-fold increase in imidacloprid residue in dried tea. It also undermines the cell wall structure of tea leaves, resulting in a 12–18% decrease in the cell breaking rate during the rolling process, consequently affecting the density of the tea strands [67].

Heavy metals influence tea quality by competing for ions and inhibiting enzyme activity. Cadmium binds competitively to the cofactor sites of chlorophyll synthase, resulting in an 18–22% reduction in the chlorophyll a/b ratio and a 25–30% decline in the net photosynthetic rate of tea plants. Exceeding the standard copper content (>30 mg/kg) activates phenylalanine ammonia lyase (PAL) activity. While the total quantity of tea polyphenols may rise by 15–20%, this results in an imbalance in catechin proportions, specifically an 8–12% reduction in ester catechin levels, thereby altering the characteristics of the tea infusion. Pollution from processing equipment is a significant concern. Copper debris resulting from the prolonged wear of copper rolling equipment can elevate the copper content in dry tea to over 30 mg/kg, surpassing the national standard limit by 15%. The use of stainless steel processing tools can mitigate this type of pollution by 60% through physical isolation [62]. To minimize the influence of heavy metals on tea quality, it is essential to define the acceptable control limits of heavy metals in the soil. Table 3 outlines the differences in limit standards for core heavy metals between tea-specific soil and general agricultural soil.

### 3.2. Hazards and Effects on the Human Body

The deleterious effects of pesticide residues exhibit a significant dose–response correlation. Acute exposure to organophosphate insecticides, such as oxytetracycline, leads to the suppression of acetylcholinesterase activity [68], which leads to an excessive accumulation of acetylcholine at neural synapses. This accumulation manifests as muscarinic symptoms, such as salivation and pupil constriction, as well as nicotinic symptoms, including muscle tremors and respiratory failure [63]. Chronic ingestion can lead to oxidative stress and DNA damage; for instance, pyrethroid pesticides may disrupt cellular calcium channels, thereby elevating the risk of neurodegenerative diseases [69]. The International Agency for Research on Cancer (IARC) has classified glyphosate as a Class 2A probable carcinogen. Moreover, its metabolite, aminomethylphosphonic acid, may indirectly promote cancer by modifying the balance of gut flora.

The toxicity of heavy metals is intricately linked to their chemical forms. Methylmercury can penetrate the central nervous system via the blood–brain barrier and interact with glutathione, resulting in a 30–40% increase in the apoptosis rate of hippocampal neurons, which contributes to memory decline and motor coordination disorders [70]. Cadmium impairs the mitochondrial function of renal tubular epithelial cells by substituting for calcium at binding sites. Prolonged exposure can elevate urinary β_2_-microglobulin levels by 2–3 times, signifying early renal injury [71]. Lead interacts with hydroxyapatite to form persistent compounds that limit osteoblast activity and promote osteoclast development. This process results in a 10–15% decrease in bone density and an increased risk of fractures.

### 3.3. Hazards and Impacts on Ecosystems

Pesticide residues present ecological risks via the migration and diffusion across various media. The combined exposure to neonicotinoids and triazole pesticides, at environmental concentrations of 0.89–1.62 μg/L, results in a 40–60% reduction in the population density of Daphnia magna. This toxic effect can persist across generations, specifically observed in F12, characterized by an extended reproductive cycle and diminished reproductive capacity. Glyphosate induces a soil microecological imbalance by reducing nitrogen-fixing bacteria populations by 30% to 50% through inhibition of the shikimic acid pathway. Additionally, it alters fungal community structures, leading to a 25% reduction in the colonization rate of arbuscular mycorrhizal fungi in the rhizosphere of tea trees [63]. Its residues can elevate the total concentrations of hexachlorocyclohexane and DDT in soil to levels approaching or exceeding the safe control value of 0.10 mg/kg, while the content of benzo[a]pyrene may approach the critical standard of 0.55 mg/kg. This situation further deteriorates soil nitrogen and phosphorus cycling efficiency and contributes to functional degradation. Pesticides, including imidacloprid, present in tea garden runoff can adversely affect aquatic ecosystems. Even when concentrations comply with national standards (≤10 mg/kg), they may surpass local ecological thresholds, resulting in a 15–20% reduction in the aquatic insect diversity index [72].

Heavy metals exhibit considerable biomagnification effects, contributing to ecological toxicity. Lead contributes to soil functional degradation by decreasing urease and phosphatase activities by 20–25% and 15–20%, respectively, through its binding to the active sites of soil enzymes, which results in reduced nitrogen-phosphorus cycling efficiency [73]. Under cadmium stress, the secretion of organic acids, including citric acid, by tea tree roots increased by 30–40%. This alteration affected the rhizosphere pH, inhibiting mycorrhizal fungal infection and leading to a 15–20% reduction in nutrient absorption efficiency [74]. A reduction in rhizosphere pH facilitates the desorption of cadmium ions; generally, when soil pH is less than or equal to 6.5, the acceptable cadmium concentration should be maintained at or below 0.30 mg/kg, while it may be increased to 0.40 mg/kg when pH exceeds 6.5. Fluoride levels must not exceed 1200 mg/kg in acidic soil and 1500 mg/kg in alkaline soil. The acidic rhizosphere environment will further elevate the available cadmium content, intensifying the dual stress on rhizosphere microorganisms. Cadmium in tea exhibits a biological enrichment factor (BCF = 0.3–0.6), leading to a concentration increase of 2–3 times in herbivorous insects. This accumulation is subsequently transferred to higher trophic levels, resulting in cadmium concentrations in top predators that exceed 5–8 times the initial levels.

## 4. The Development and Application of Biosensors

Biosensor technology has emerged as a crucial instrument for detecting heavy metals and pesticide residues in agricultural products, driven by the growing demand for food safety and environmental pollution monitoring. Its advantages include high sensitivity, excellent selectivity, and rapid response times. This section reviews the performance indicators (Table 4) and recent advancements in mainstream biosensors, including fluorescence, surface-enhanced Raman spectroscopy, colorimetric methods, surface plasmon resonance, electrochemistry, and piezoelectric sensors. The study examines the sensing mechanism, material innovation, and application performance in real samples, including tea, fruits, and vegetables. This work offers a theoretical foundation and technical guidance for the selection and optimization of sensors across various scenarios.

### 4.1. Fluorescence Biosensor

Fluorescent biosensors typically utilize fluorescent dyes, quantum dots, and nanomaterials as signal sources to detect heavy metals and pesticide residues through the specific recognition of biological materials, including antigen–antibody interactions, aptamers, and cells. Their advantages include high sensitivity, rapid response, elevated throughput, minimal background signal, and straightforward automation. As summarized in Table 4, these sensors consistently demonstrate excellent analytical performance in tea samples, with wide linear ranges (e.g., 0.01–10 μg/L), high precision (RSD ≤ 5.0%), and reliable accuracy reflected in spiked recovery rates of 88.2–102.5%.

#### 4.1.1. Fluorescent Biosensor Based on the “Sign On” Strategy

Quantum dots (QDs, e.g., Sigma-Aldrich, St. Louis, MO, USA), upconversion nanoparticles (UCNPs, e.g., NaYaTech, Turin, Italy), and carbon quantum dots (CDs, e.g., ACS Material, Pasadena, CA, USA) represent the predominant nanofluorescent materials utilized in this category of sensors. Chen et al. [81] developed a fluorescence resonance energy transfer (FRET) sensor that integrates UiO-66-NH_2_ (e.g., Sigma-Aldrich, St. Louis, MO, USA) with an aptamer. The sensor employed the fluorescence quenching characteristics of metal–organic frameworks (MOFs) and the selective identification of Cd^2+^ by the aptamer, achieving linear detection within the range of 0.01–10 μmol/L, with a detection limit of 0.01 μmol/L. Optimizing the aptamer concentration at 200 nmol/L and UiO-66-NH_2_ mass concentration at 0.2 mg/mL resulted in a spiked recovery rate of 85.7–103.2% in rice samples, with a relative standard deviation of less than 7.6%. The response mechanism involves Cd^2+^-induced dissociation of the aptamer from the UiO-66-NH_2_ surface, which restores the fluorescence signal of the 6-FAM labeled aptamer (excitation/emission wavelength 495/518 nm), demonstrating a consistency coefficient of R^2^ = 0.921 when compared to high-performance liquid chromatography (HPLC). Modifying the aptamer sequence allows for the detection of additional heavy metals, establishing a novel approach for on-site, real-time monitoring of cadmium contamination in tea and tea garden soil.

Wang et al. [82] developed a dual-signal ratiometric fluorescence sensor utilizing TPE-Fc and GSH-AuNCs, integrating it with an acetylcholinesterase catalytic system for the detection of organophosphorus pesticides. The linear range was 10 to 2000 ng/mL, with a detection limit of 2.05 ng/mL. The sensor amplifies cyan fluorescence at 472 nm via TPE-Fc oxidation facilitated by H_2_O_2_, while ·OH causes orange fluorescence quenching of GSH-AuNCs at 615 nm, enabling visual detection through fluorescence color alterations. The accompanying WeChat mini program (WeChat, Version 8.0; Tencent, Shenzhen, China), integrated with smartphone RGB recognition technology, attained a spiked recovery rate of 85.7–103.2% in cucumber and lettuce samples, thereby offering technical assistance for intelligent on-site screening of organophosphorus pesticide residues in tea.

Li et al. [83] developed a fluorescent sensor, TTB, derived from oligothiophene, achieving a detection limit of 0.23 μM for Hg^2+^, a response time of less than 1 min, and demonstrating excellent selectivity and resistance to interference. The method has been effectively utilized for the quantitative analysis of Hg^2+^ in various samples, including water, soil, tea, and seafood, as well as for fluorescent imaging of living plants like Arabidopsis thaliana and onion. The response mechanism, grounded in Schiff base coordination, has been substantiated through Job’s plot, FTIR, and density functional theory (DFT, e.g., Gaussian 16, Revision C.01; Gaussian, Inc., Wallingford, CT, USA), offering an effective technical solution for the rapid visual detection of Hg^2+^ in tea.

Wang et al. [84] conducted a review on the use of fluorescent covalent organic frameworks (FCOFs) for heavy metal ion sensing. They systematically analyzed the synthesis strategies for boron-based, imine-based, triazine-based, and other FCOFs. Additionally, they utilized density functional theory (DFT) to elucidate fluorescence mechanisms, including photo-induced electron transfer (PET) and resonance energy transfer (RET). Their application efficacy in identifying harmful metal ions, such as Hg^2+^ and Cd^2+^, was encapsulated, reaching detection limits as low as nanomolar quantities. Their increased selectivity and anti-interference properties in actual samples, such as soil, water, and tea, provide valuable insights for the material design of FCOFs in detecting heavy metals in tea.

#### 4.1.2. Fluorescent Biosensor Utilizing the “Sign Off” Strategy

This sensor type fundamentally operates by quenching or significantly diminishing the fluorescence signal in the presence of the target, while the fluorescence intensity stays heightened in its absence. Feng et al. [85] created a molecularly imprinted polymer (MIP) ratiometric fluorescence sensor utilizing carbon dots (CDs) and SiO@CdTe@MIP. The synergistic interaction of these materials facilitates the detection of bisphenol A (BPA), employing CDs as a reference signal to improve detection stability. This sensor has been effectively utilized for the accurate identification of BPA in water samples. This sensor’s high selectivity, in conjunction with molecular imprinting technology and the superior fluorescence performance of quantum dots, effectively mitigates complex matrix interference.

Guo et al. [86] synthesized green fluorescent graphite nitride carbon nanoparticles (CNNPs) through a one-pot solvothermal method, serving as a self-calibrating signal source, and prepared red fluorescent cadmium telluride sulfide quantum dots (CdTe_0.16_S_0.84_, QDs) via a redox method. The researchers developed a ratiometric fluorescence sensor utilizing CNNPs-CdTe_0.16_S_0.84_, with quantum dots serving as the response signal source, for the detection of Hg^2+^ ions. The sensor exhibits a linear range of 0.05–25 nM and a detection limit of 0.009 nM. The sensitivity to Hg^2+^ is markedly superior to that of Cu^2+^ and Ag^+^. Analysis using the Stern-Volmer equation and DFT confirms that this sensitivity difference arises from electron transfer and electrostatic interactions between Hg^2+^ and the CdTe_0.16_S_0.84_ QDs. The advanced smartphone-assisted hydrogel fluorescence sensor offers a novel approach for the portable on-site detection of Hg^2+^ in environmental water and tea.

Huang et al. [87] developed an ADA-UCNPs-aptamer fluorescence sensor utilizing magnetic separation technology for the detection of thiamethoxam. Functionalization of magnetic nanoparticles with aptamers, followed by base pairing with cDNA-UCNPs, led to thia-methoxam binding, which triggered the detachment of UCNPs and resulted in a decrease in fluorescence at 544 nm for quantification purposes. The sensor exhibits a detection limit of 0.08 ng/mL and a linear range spanning from 0.4 to 102.4 ng/mL. Magnetic separation technology decreases detection time to 25 min. The recovery rate for spiked cucumber and cabbage samples varied from 82.67% to 109.33%, with a relative standard deviation of ≤10.68%. The detection limit surpasses that of enzyme-linked immunosorbent assay at 0.12 ng/mL, and magnetic separation efficiency has improved threefold. This method is appropriate for the concurrent detection of various pesticide residue components in agricultural products, including tea.

It is essential to recognize that while fluorescent sensors have significant sensitivity and controllability, certain systems include complex signal response mechanisms, vulnerability to fluorescent dye bleaching, biological toxicity of nanomaterials, and unstable chemical properties. In the future, it is essential to improve sensing performance by optimizing experimental methodologies and developing innovative fluorescent probes.

### 4.2. Surface Enhanced Raman Spectroscopy

Raman spectroscopy [88] enables the identification of multiple analytes through the collection of molecular vibrations, facilitating “molecular fingerprint recognition” of their composition. This method has been utilized for the sensitive detection of various analytes, including small molecules and proteins. Raman spectroscopy records the intrinsic vibrational fingerprint of a sample, whereas SERS [89] utilizes nanostructured noble-metal substrates to boost the Raman signal by factors ranging from 10^6^ to 10^11^, facilitating straightforward operation, rapid reaction times, and elevated sensitivity and specificity (e.g., LOD of 0.05 μg/L for imidacloprid), along with label-free detection and improved stability. The performance data shown in Table 4 validate its effectiveness in intricate matrices such as tea, demonstrating respectable recoveries nearing 100% and commendable repeatability(RSD ≈ 6.2%).

Pesticide detection in current SERS sensors remains challenging due to biological interference within complex food matrices. 4-mercaptobenzene (MBN) exhibits a prominent Raman peak, effectively mitigating optical interference from biomolecules in food matrices, and is frequently utilized as a Raman probe in SERS sensors. A competitive immunosensor for detecting imidacloprid was developed using bimetallic gold-silver nano rectangular prisms (AuNR@Ag). This system involved the attachment of fixed antigens and MBN as Raman probes, alongside Fe_3_O_4_ magnetic nanoparticles (e.g., Micromod Partikeltechnologie GmbH, Rostock, Germany) functionalized with imidacloprid antibodies serving as signal enhancers. Imidacloprid competes with fixed antigens for binding to particular antibodies, resulting in a decrease in the quantity of MBNs that produce Raman spectroscopic signals. The approach has been successfully used for quantifying imidacloprid in actual samples, exhibiting a linear range of 10–400 nmol/L, a detection limit of 9.58 nmol/L, and attaining near-quantitative recovery (≈100%).

The use of MBN as a Raman tag significantly reduces interference from organic contaminants, while Fe_3_O_4_ magnetic nanoparticles increase the loading capacity of the Raman probe, thereby optimizing the separation process. The intricacy of pesticide application requires the identification of several pesticides to more adequately meet human demands than the identification of a singular pesticide. Song et al. [90] utilized biosensors incorporating Au@Ag core–shell nanorods (Au@Ag NRs) to amplify Raman signals and determine aptamer specificity. 4-Mercaptobenzoic acid (4-MBA) and aptamer-modified Au@Ag nanorods (NRs) serve as Raman signal probes, adsorbing onto the surface of Au@Ag NRs in the absence of bacteria within the reaction system. Following the introduction of Salmonella typhimurium, the aptamer selectively identifies and binds to the cells. The decrease in free ligands in the solution reinstated the Raman spectroscopy “hotspot” between Au@Ag NRs and amplified the Raman signal of 4-mercaptobenzoic acid (4-MBA). A quantitative analysis of Salmonella typhimurium was performed utilizing Raman enhanced spectroscopy.

Liu et al. [88] developed Ag-covered ‘’hedgehog-shaped” nanosphere arrays (Ag/HLNAs) for Raman spectroscopy through a process of self-assembly and reactive ion etching. The enhancement factor achieved was 2.79 × 10^7^, with a detection limit for Rhodamine 6G at 10^−10^ M and a relative standard deviation of less than 10%. The linear detection range for pesticide Fumeishuang was established between 10^−8^ and 10^−4^ M, with a R^2^ value of 0.954. The detection limit was determined to be 10^−8^ M. This device exhibits high sensitivity and uniformity, making it suitable for the precise detection of trace amounts of organophosphorus and other agricultural residues in tea via Raman spectroscopy. It offers technical support that combines stability and practicality for on-site rapid screening.

Terry et al. [91] created a Raman spectroscopy nano-substrate using bio-waste-derived nano-cellulose alloy nanoparticles alongside a portable Raman instrument. This setup enabled the sensitive detection of carbaryl and thibendazole, achieving detection limits of 1.34 and 1.01 mg/L, respectively, both below the recommended concentration for agricultural use. The detection signals of thibendazole spray solution and droplets are consistent, facilitating the identification of commercial pesticides.The sustainable and reproducible Raman spectroscopy detection system offers a systematic approach for the swift assessment of agricultural residues in tea, oversight of spray residues, and the enhancement of eco-friendly detection technologies.

Raman spectroscopy sensors show promise for rapid pesticide analysis and identification; yet, current research is still in its nascent stages, encountering several obstacles. Raman spectroscopy demonstrates restricted selectivity for homologous molecules with similar structural characteristics and Raman activity, hindering the concurrent study of various pesticide residues. The Raman signal from agricultural product matrices often coincides with the spectra of pesticide molecules, leading to considerable matrix effects. Furthermore, some pesticides pose difficulties for detection by direct testing owing to their structural complexity.

**Figure 5:**
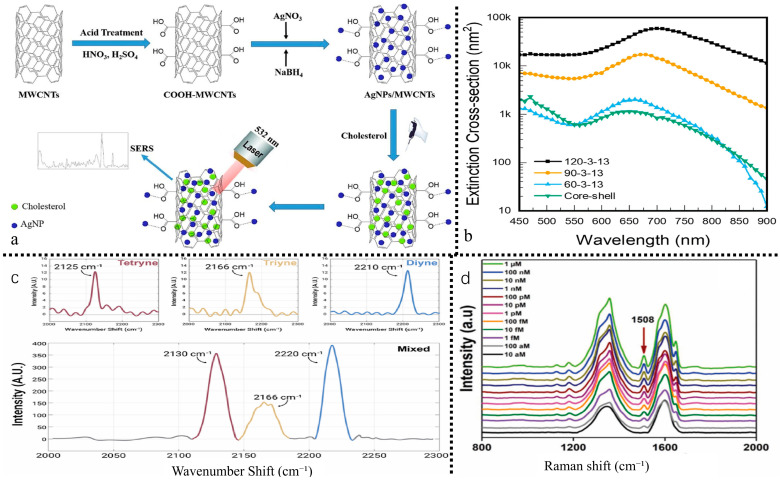
(**a**) Detection mechanism of Surface-Enhanced Raman biosensor utilizing silver nanoparticles and multi-walled carbon nanotubes [93], (**b**) The extinction cross-section of different configurations in a wavelength range from 450 nm to 900 nm [94], (**c**) G-SERS spectra of different concentrations of target-DNA ranging from 1 μM to 10 aM [95], (**d**) SERS analysis of Raman tagged antibodies for EV biomarker detection [96].

Surface-Enhanced Raman biosensor (SERS) [92] utilizes molecular vibrations on rough metal surfaces or nanostructures to produce distinctive fingerprint spectral signal intensities, enabling the determination of molecular concentration. This method offers high sensitivity, low cost, and rapid performance. The Raman substrate and Raman signal molecules are primary determinants influencing surface-enhanced raman signals. High-performance Raman substrates must exhibit extensive areas of high-density hotspots, superior uniformity, reliable reproducibility, and elevated enhancement factors. Dikmen et al. [93] demonstrated the synthesis of a hybrid material through the attachment of Ag metal nanoparticles to the surface of multi-walled carbon nanotubes (MWCNTs). The hybrid material was utilized as a SERS substrate for the adsorption of cholesterol molecules on its surface (Figure 5). In SERS research, silver nanoparticles integrated into hybrid materials amplify Raman signal strength by electromagnetic enhancement methods. Simultaneously, MWCNTs enhance SERS intensity via chemical enrichment and promote hotspot formation by arranging silver nanoparticles in a dense and systematic manner. This configuration enhances SERS signal intensity, allowing the rapid and dependable detection of very low cholesterol amounts.

Zhang et al. [97] developed a simple and sensitive SERS sensor for detecting benzo[a]pyrene in food, utilizing gold nanostars@reduced graphene oxide (AuNS@rGO). The detection strategy involves adsorbing benzo[a]pyrene onto reduced graphene oxide, followed by SERS detection of the adsorbed molecules. The substantial electric field produced by gold nanostars during laser irradiation significantly enhances the Raman signal of benzo[a]pyrene, resulting in heightened sensitivity to the target analyte. The SERS sensor exhibits a broad linear detection range for benzo[a]pyrene under optimized conditions.

He et al. [92] developed a dual enhanced SERS sensor utilizing graphene and gold-silver hybrid nanostructures. The combined effects of electromagnetic enhancement (EM) and chemical enhancement (CM) resulted in an enhancement factor of 3.85 × 10^8^. The detection range for Pb^2+^ was established between 10 pM and 100 nM, with a detection limit of 4.31 pM. The material demonstrates the potential for reuse on a minimum of three occasions, exhibiting significant specificity and stability within complex aqueous samples. The integration of the sensor with FDTD simulation validated the sensing mechanism, offering a technical reference of both theoretical and practical significance for the ultra-sensitive detection, reusability, and adaptability to complex substrates of heavy metals in tea through Raman spectroscopy sensors.

Qi et al. [98] developed a polycytosine (polyC) mediated surface enhanced Raman scattering (SERS) nanolabel sensor. The coordination of thymine with Hg^2+^ (T-Hg^2+^-T) facilitates rapid amplification of the SERS signal via nanolabel aggregation. PolyC enhances Raman activity via coordination with silver cytosine and regulation of length. The sensor exhibits a detection range of 0.1–1000 nM for Hg^2+^ and demonstrates good selectivity towards other metal ions. This detection system, characterized by high sensitivity and specificity, introduces a novel design concept and application reference for the accurate identification and rapid screening of heavy metals, including Hg^2+^, in tea through the use of Raman spectroscopy sensors.

Chen et al. [99] developed a nanolabel sensor for surface-enhanced raman scattering (utilizing polycytosine (polyC) as a mediator. The coordination of thy-mine-Hg^2+^-thymine (T-Hg^2+^-T) facilitates rapid amplification of the SERS signal via nanolabel aggregation. PolyC enhances Raman activity via silver-cytosine coordination and length regulation. The sensor exhibits a detection range of 0.1–1000 nM for Hg^2+^ and demonstrates strong selectivity for other metal ions. This detection system, distinguished by its high sensitivity and specificity, presents an innovative design idea and acts as a reference application for the precise identification and fast screening of heavy metals, such as Hg^2+^, in tea using Raman spectroscopy sensors.

### 4.3. Colorimetric Biosensors

The colorimetric sensor operates on the principle of colorimetric reaction, which induces color changes via the interaction between the target substance and the chromogenic reagent for qualitative and quantitative analysis. It has the advantages of being visually discernible, intuitive, and rapid in detection. Gold nanoparticles (AuNPs) and silver nanoparticles (AgNPs) serve as the primary materials for this sensor type because to their extensive specific surface area, elevated extinction coefficient, superior biocompatibility, and peroxidase-mimetic characteristics. Table 4 shows that colorimetric tests have high sensitivity (LOD of 0.005 μg/L for Hg^2+^) and reliable quantitative performance in tea and ambient water samples. They also have outstanding recovery effectiveness and accuracy (RSD = 3.8%).

Kaur et al. [100] developed a colorimetric detection method for the organophosphate pesticide methyl phosphate: Hydrogen peroxide (H_2_O_2_) produced through choline oxidase (ChOx)-mediated hydrolysis is oxidized by horseradish peroxidase (HRP), leading to colorimetric alterations in the sensitive dye ABTS. Methyl phosphate impedes ChOx activity in the presence of phosphorus oxide, thereby diminishing the colorimetric signal and facilitating the indirect quantification of pesticide residues.

Shayesteh et al. [101] developed a colorimetric biosensor for the detection of aflatoxin B1 (AFB1) by functionalizing AuNPs with polyA aptamers (polyA apts): AuNPs/polyA apts exhibit resistance to aggregation produced by the cationic polymer PDDA, hence preserving a red dispersed state at 520 nm. In the presence of AFB1, the aptamer selectively attaches and undergoes conformational folding, while PDDA promotes the aggregation of AuNPs, resulting in a shift in the absorption peak to 620 nm (blue). AFB1 is measured using the A620/520 ratio, exhibiting a linear range of 0.5–20 ng/mL and a detection limit of 0.09 ng/mL. It has been effectively utilized for the detection of corn samples.

Xie et al. [102] examined the utilization of multi-color colorimetric sensors employing gold nanomaterials for food hazard detection. They manipulated the morphology of gold nanomaterials via etching and growth techniques, leveraged local surface plasmon resonance properties to induce color variations, and integrated signal amplification methods to attain detection sensitivity at the pg/mL level. This can be utilized for the identification of pesticides and heavy metals, such as Hg^2+^ and Cr^6+^, offering material design guidelines for the visual fast screening of tea contaminants.

Hu et al. [103] developed a Fe_3_O_4_@MnO_2_ core–shell nanostructured colorimetric sensor for the simultaneous detection of Hg^2+^ and Cu^2+^ in tea leaves. The MnO_2_ shell exhibited peroxidase-like activity that catalyzed the coloring of 3,3′,5,5′-tetramethylbenzidine (TMB), whilst the Fe_3_O_4_ core improved stability via magnetic separation. The core–shell architecture enhanced catalytic efficiency by over thrice. The sensor’s detection limits for Hg^2+^ and Cu^2+^ are 0.12 nM and 0.25 nM, respectively. The approach exhibits an almost quantitative spiking recovery (≈100%) in tea samples, with recoveries between 95.2% and 103.7%, and a relative standard deviation below 5%. Setting the pH to 5.5 and the temperature to 37 °C minimizes substrate interference.

The main drawbacks of current colorimetric sensors include poor adaptability to complex matrices, high prices of specific reagents, and inadequate detection stability. Further progress is necessary via material modification and methodological innovation.

### 4.4. Surface Plasmon Resonance Sensor

Surface-plasmon resonance (SPR) [104] sensors operate on the principle of resonance angle or wavelength shift induced by variations in the refractive index of the sensing media interface for detecting purposes. They have advantages of cheap cost, little time investment, and high accuracy, and have increasingly been used in the fields of pesticide and heavy metal detection. Microfluidic chip technology enables the integration of “preprocessing-detection,” providing an efficient platform for multi-target detection. The key performance metrics in Table 4 underscore its efficacy in tea and soil analysis, exhibiting exceptional, virtually total recoveries and consistent findings (RSD = 5.3%).

Zhang et al. [105] integrated local surface plasmon resonance (LSPR) nanosensors with microfluidic chips to facilitate in situ ultra-sensitive detection of Hg^2+^ utilizing dark field microscopy spectroscopy. The sensor employs a unique oligonucleotide-functionalized core-satellite nanostructure as an image and identification probe, achieving a detection limit of 2.7 pM and a broad linear range, so offering a technical solution for the quick in situ detection of Hg^2+^ in tea.

Zhong et al. [106] developed an ion-imprinted chitosan-modified LSPR sensor, wherein gold nanoparticles were affixed to the surface of a multimode optical fiber to induce LSPR. A Ni^2+^-imprinted chitosan coating was fabricated using the dip-coating method. EDTA treatment resulted in the formation of specific holes for Ni^2+^ recognition; with four layers of coating, sensitivity attained 185 pm/μM and the detection limit was 0.512 μM. The selectivity for Ni^2+^ is markedly superior to that of pure chitosan, and it demonstrates the capability for reuse up to six times. This method is effective for the selective detection of Ni^2+^ in tea.

Bakhshpour et al. [107] conducted a comparative analysis of the detection performance of SPR sensors modified with poly(hydroxyethyl methacrylate) film, polymer nanoparticles, and gold nanoparticles for Cd^2+^ detection. The sensor incorporating nanoparticle signal amplification demonstrated a detection limit of 0.01 μg/L, which is below the WHO standard of 3 μg/L. It exhibited a linear range of 0.01–50 μg/L and showed superior selectivity for Cd^2+^ compared to Cr^2+^, Pb^2+^, and Zn^2+^. The response time ranged from 6 to 7 min, and stability was assessed as satisfactory. This method is applicable for detecting wastewater and tea samples.

Çakır et al. [108] developed a molecularly imprinted nanofilm-modified SPR sensor for the detection of dimethoate and furan, achieving detection limits of 8.37 ng/L and 7.11 ng/L, respectively, which are notably lower than those of LC-MS/MS methods (16.92 ng/L, 20.47 ng/L). The recovery rate ranged from 90% to 95% for concentrations between 50 and 1000 ng/L. The molecularly imprinted membrane exhibited superior selectivity compared to the non-imprinted membrane, offering an effective approach for detecting trace pesticides in tea.

The main constraint of SPR sensors is their adaptation to intricate surroundings; yet, their sensitivity is enough for trace detection, along with acceptable stability and specificity, fulfilling the detection needs for pesticides such as neonicotinoids.

### 4.5. Electrochemical Biosensors

Electrochemical biosensors use fixed electrodes as substrates to detect target chemicals via the particular identification of bioactive compounds. They transform biological concentration signals into electrical signals, including current, resistance, and potential for detection. Sensitivity is often improved by the use of techniques like nanomaterial amplification and hybrid chain reactions, which provide elevated sensitivity, fast analysis, cost-effectiveness, and operational simplicity. It is classified into two categories: label-free and labeled.In accordance with the assessments presented in Table 4, these sensors demonstrate exceptional efficacy in real-sample analysis (tea, fruits, vegetables), attaining highly accurate quantitative recoveries and notable precision (RSD = 4.5%), highlighting their appropriateness for on-site, portable quantitative detection.

#### 4.5.1. Label Free Electrochemical Biosensor

This sensor detects changes in electrical impulses caused by the development of analyte-target complexes. Electrochemical impedance spectroscopy (EIS) is a conventional method in which the attachment of probe molecules to the electrode surface modifies features including interface capacitance and double-layer capacitance, resulting in changes in impedance signals [109,110]. Gold (e.g., NanoComposix, San Diego, CA, USA), silver, and platinum nanoparticles serve as substrates for signal amplification.

Madianos et al. [111] l laterally deposited platinum nanoparticles between forked electrodes (IDEs) and developed an impedance sensor for the detection of imidacloprid by securing the adapter with gold-sulfur bonds. The binding of imidacloprid to the adapter impeded electron transfer, leading to an increase in impedance and enabling quantification. The detection limit reached 1 pmol/L, demonstrating significantly improved sensitivity compared to the bare gold electrode sensor.

Xu et al. [112] and Ali et al. [113] developed highly porous gold (HPG)-based label-free impedance aptamer sensors for the detection of imidacloprid. HPG was synthesized through electrodeposition and subsequently self-assembled with imidacloprid aptamer. Detection was accomplished through the measurement of changes in electron transfer resistance resulting from ligand-target binding, exhibiting a linear range of 0.5–300 nmol/L and a detection limit of 0.34 nmol/L. The method exhibits high selectivity, reproducibility, and stability, and has been effectively utilized for the detection of pesticides in fruits and vegetables, offering a viable approach for identifying similar pesticides in tea.

Furthermore, differential pulse voltammetry (DPV), cyclic voltammetry (CV), and square wave voltammetry (SWV), among others, are often used for the electrical signal analysis of label-free sensors, therefore expanding the range of detection application.

#### 4.5.2. Label Type Electrochemical Biosensor

This sensor utilizes fluorescent dyes, enzymes, metal ions, quantum dots, and analogous substances as signal labels, generating changes in electrical signals via the formation of probe-target complexes, often demonstrating increased sensitivity and versatility. To tackle the problem of insufficient immunogenicity of small molecules in pesticides, competitive methods are often used to create detection systems. Pérez-Fernández et al. [114] designed a monoclonal antibody-based electrochemical sensor, integrating a screen-printed carbon electrode (SPCE) with a competitive immunoassay for the detection of imidacloprid (IMD). The sensor demonstrated excellent repeatability (RSD = 9%), exhibiting a logarithmic response within the range of 50–10,000 pM and a detection limit of 24 pM, which is below the regulatory threshold. The response range and sensitivity surpassed those of HPLC-MS/MS and ELISA, while the matrix effect in tap water samples was below 6.5%, proving it appropriate for anti-interference detection of imidacloprid in tea.

Yang et al. [115] developed an amino-modified metal–organic framework, UiO-66-NH_2_, which functions as an electrochemical sensor with a specific surface area of 1018 m^2^/g and adsorption capacities for Cd^2+^ and Pb^2+^ of 230 mg/g and 271 mg/g, respectively. Utilizing these two ions as labels, in conjunction with an immune response and square wave voltammetry (SWV), they achieved synchronous detection of triazophos and thiacloprid, exhibiting linear ranges of 0.2–750 ng/mL and detection limits of 0.07 ng/mL and 0.1 ng/mL, respectively, with corresponding signal potentials of −0.82 V (Cd^2+^) and −0.56 V (Pb^2+^). This sensor offers a technical reference for optimizing the synchronized sensitive detection and tagging technique of multi-component pesticide residues in tea by a labeled electrochemical biosensor.

Zhang et al. [116] created a labeled electrochemical biosensor utilizing Fe_3_O_4_@Au core–shell nanoparticles for labeling and a hybridization chain reaction (HCR) amplification method for the detection of Ag^+^. The sensor specifically identifies target ions via C-Ag^+^-C base pairing, initiates HCR to create a super sandwich structure with ferrocene labels, and integrates with magnetic gold electrode enrichment. The detection limit is 0.5 fM, and the dynamic range is from 1 fM to 100 pM. It exhibits exceptional selectivity for Ag^+^ and demonstrates effective performance in both tap water and lake water samples. This sensor offers a theoretical framework and practical utility for the ultra-sensitive detection of heavy metals like Ag^+^ in tea through labeled electrochemical biosensors, as well as for investigating complicated substrate adaptation.

Aptamers are often used as recognition elements in labeled electrochemical biosensors because of their low cost and simplicity of modification. Electrochemical sensors [117] exhibit considerable promise for pesticide residue monitoring in tea, because to their characteristics of easy miniaturization, simple operation, and rapid, accurate testing results. However, many challenges need consideration, such as the stability of electrode surface modification materials and the interference from sugars, proteins, and other constituents in the actual sample matrix. Therefore, it is essential to develop more innovative methods for improved and accurate detection of pesticide residues.

### 4.6. Piezoelectric Biosensors

Piezoelectric biosensors use the sensing capabilities of piezoelectric quartz resonators to detect changes in resonator frequency due to interactions with target substances. They have significant benefits, such as a wide response range, high sensitivity, excellent signal-to-noise ratio, simple construction, reliable operation, and low weight.

A quartz crystal piezoelectric sensor exemplifies this domain, with its fundamental principle involving the attachment of the target receptor to the surface of the quartz crystal electrode. Upon precise binding of the receptor to the ligand, the electrode’s surface characteristics alter, resulting in a shift in the crystal’s resonance frequency. Monitoring frequency variations enables both qualitative and quantitative study of the target object. The piezoelectric sensor utilizing quartz crystal microbalance (QCM) as the transducer is the most prevalent: by altering recognition elements like antibodies, aptamers, and specific receptor proteins on the crystal’s surface, the mass variation in the surface load is transformed into a resonant frequency signal, which has been employed for detecting small molecule pollutants, including marine biotoxins. Despite the attributes of automation, user-friendliness, portability, affordability, and ease of miniaturization, the practical application of piezoelectric biosensors in marine biotoxin detection remains constrained by challenges including prolonged baseline establishment time, reliance on novel technological support, and elevated instrument costs.

Fathizadeh et al. [118] validated the capabilities of piezoelectric biosensors in miniaturization and cost-effectiveness, and developed sensing devices utilizing the piezoelectric characteristics of DNA. Testing revealed that the current-voltage spectra of poly(CG) had distinct peaks under varying external forces (peak at 0.8 V at F = 0.3 pN, peak at 3.4 V at F = 0.01 pN), and its electrical response pattern was predictable by multifractal analysis. This study offers essential parameters for optimizing sensor performance and establishes a foundation for the system design and theoretical investigation of piezoelectric biosensors in the detection of pesticide residues and heavy metals in tea.

Skládal’s [103] study further substantiated the utility of piezoelectric biosensors for pesticide residue detection: an immunosensor based on the QCM principle was developed for label-free real-time detection of carbaryl via the phase shift method, achieving a detection limit of 0.14 ng/mL with a 9 MHz resonator and 11 ng/mL with a 100 MHz resonator; the integration of competitive immunoassay formats enables highly sensitive detection of small molecule pesticides, while also offering advantages such as rapid response, reusability, and cost control. This study offers a theoretical foundation and technical guidance for the effective detection of trace pesticide residues in complex matrices, such as tea, utilizing piezoelectric biosensors.

**Figure 6:**
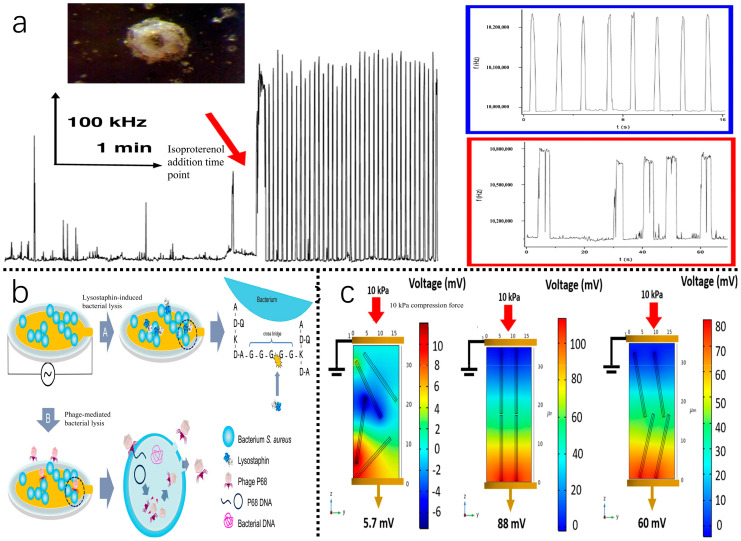
(**a**) Cardiomyocyte cluster beating behavior on a 10 MHz gelatin-modified piezoelectric crystal and the influence of β-blockers [80], (**b**) Schematic representation of the course of the experiment on the surface of the QCM-D sensor [120], (**c**) FE simulation of output voltage generated across the samples in response to a compression of 10 kPa for fibers randomly dispersed, perfectly aligned fibers and tilted fibers in NN_MF/PDMS composites [121].

The ultra-high frequency electromagnetic piezoelectric acoustic sensor (EMPAS) developed by Mészáros et al. [119] demonstrates high-sensitivity detection of biomolecules at a resonance frequency of approximately 1 GHz. This is achieved by the optimization of system setup, including interface programs and integrating features for measuring acoustic quality factors, with the increase in analytical sensitivity using 49th to 53rd order harmonics (Figure 6). The sensor produces a frequency shift of 22 kHz upon the adsorption of β-casein and efficiently monitors the surface contacts of the actin complex associated with coagulation proteins. Analyzing high-order harmonics, particularly the zero-crossing points of the third harmonic, enables the accurate determination of resonance frequencies, providing an effective approach for real-time, label-free biomolecule identification. Technological developments in surface adsorption analysis and improvements in sensitivity provide a scientific basis for expanding piezoelectric biosensors to detect biomolecules in tea.

### 4.7. Innovative Sensing Techniques and Microfluidic Integration

Advancements in biosensing technology are evident in the enhancement of signal transduction processes and the transformation of recognition components, along with their thorough integration into microsystem platforms. Recent research has concentrated on creating innovative biorecognition components, like DNAzymes and peptide aptamers, to overcome the constraints of conventional antibodies and enzymes regarding stability, cost, and target specificity. Simultaneously, microfluidic technology, characterized by its exact fluid control, minimal reagent use, and highly integrated “sample-in-answer-out” functionality, offers an optimal platform for the swift and high-throughput screening of contaminants in intricate matrices. The collaborative integration of these two domains is steering the advancement of tea safety detection towards a new generation of intelligent sensing systems characterized by improved anti-interference capabilities, heightened sensitivity, and mobility.

Specifically, in the realm of heavy metal detection, Morrison et al. [122] successfully designed an arrayed sensor using the cross-reactivity of four DNAzymes (17E, GR-5, EtNA, NaA43) for Pb^2+^, Ca^2+^, Na^+^, and prevalent interferents (e.g., Mg^2+^, Zn^2+^). This strategy utilized the t-SNE algorithm for dimensionality reduction and analysis of response patterns, effectively identifying and quantifying specific heavy metals (e.g., Pb^2+^) and their mixtures in environmental soil samples, thereby providing an innovative “pattern recognition” solution to the traditional issue of restricted DNAzyme selectivity. To improve detection sensitivity and applicability, Wu et al. [123] developed a hydrogel optofluidic microcavity senso. This sensor incorporates an aptamer-functionalized hydrogel film within a whispering-gallery-mode optical microcavity. The infiltration of target heavy metal ions, such as Pb^2+^ and Hg^2+^, into the hydrogel network induces a swelling change, resulting in a redshift in the microcavity’s resonance wavelength. This mechanism facilitates ultrasensitive and label-free detection of trace heavy metals, demonstrating exceptional performance in complex real-world samples, including herbal extracts. IA thorough review by Jiang et al. [124] discusses advancements in microfluidic methods for the quick screening of pesticide residues, including pyrethroids, carbamates, organophosphates, and organochlorines. The study emphasizes the benefits of merging microfluidic devices with various readout modalities (colorimetric, fluorescence, electrochemical) and delineates the considerable potential of incorporating 3D printing and nanomaterials for economical device customisation and improved performance. These innovative works provide a viable approach to enabling on-site, real-time, and intelligent monitoring of various pollutants throughout the full “tea garden-to-cup” process via the integration of new identification materials and microsystems engineering technologies.

### 4.8. Technical Comparison, Challenges, and Translational Prospects

Various biosensor types provide unique performance attributes and application possibilities for identifying pollutants within the intricate tea matrix, due to their differing recognition methods and signal transduction principles (Table 5). Fluorescent sensors rs [81] may attain ultra-high sensitivity in the nM to pM range; however, their photophysical stability is vulnerable to photobleaching, and naturally occurring fluorescent compounds in tea (such as chlorophyll and polyphenols) can lead to considerable background interference.Surface-enhanced Raman spectroscopy (SERS) [92] provides a distinctive “fingerprint” identification capability, demonstrating significant potential for the detection of pesticide molecules. Nonetheless, the signal strength and repeatability are significantly influenced by the uniformity and density of “hot spots” in precious metal nanostructures, presenting considerable hurdles for standardized production and cost management. Electrochemical sensors [111], recognized for their facile downsizing, economical detection costs, and quick reaction times, are well suited for point-of-care testing (POCT) applications. Surface-enhanced Raman spectroscopy (SERS) provides a distinctive “fingerprint” identification capability, demonstrating significant potential for the detection of pesticide molecules. Nonetheless, the signal strength and repeatability are significantly influenced by the uniformity and density of “hot spots” in precious metal nanostructures, presenting considerable hurdles for standardized production and cost management. Electrochemical sensors, recognized for their facile downsizing, economical detection costs, and quick reaction times, are well suited for point-of-care testing (POCT) applications. However, their electrode surfaces are susceptible to irreversible fouling or passivation in tea extracts rich in polyphenols and alkaloids, resulting in signal attenuation and reduced longevity. Colorimetric sensors [100] provide the most intuitive output; yet, their sensitivity and quantitative accuracy sometimes pose limitations. Despite the capability of surface plasmon resonance (SPR) [104] and piezoelectric sensors to provide label-free, real-time observation of molecular interactions, their intricate design, elevated cost, and limited throughput hinder their extensive use outside laboratory environments.

Transforming high-performing sensor prototypes from the laboratory into dependable detection instruments for tea plantations, processing facilities, and border checkpoints necessitates surmounting considerable challenges in the transition from scientific innovation to practical implementation. The main technological impediments mostly arise from the intricacy of the tea matrix [125,126]: Elevated levels of active constituents, such as tea polyphenols, can diminish fluorescence signals and induce nonspecific adsorption to sensing interfaces, while also directly inhibiting the functionality of essential biorecognition elements, including enzymes or aptamers, thus significantly undermining sensor precision and dependability. At the same time, present sensing systems have a serious deficiency in their capacity to identify several targets simultaneously. Due to the widespread co-contamination of several pesticide residues and heavy metals in tea, most sensors are restricted to detecting just single or a limited number of targets. This constraint impedes the capacity to provide a thorough safety risk assessment and fails to meet the practical need for concurrent screening of intricate pollutants in regulatory oversight. The commercialization of biorecognition elements is hindered by their degradation during storage and use, along with the difficulty of ensuring consistent performance in mass production of sensor elements. These factors are crucial for the long-term reliability, stability, and market acceptance of the final product. Moreover, each newly constructed sensing apparatus must undergo correlation validation against established national standard methodologies (e.g., GB 2763-2021, EU 396/2005) [30] and stringent regulatory compliance evaluation. This intricate and lengthy certification procedure is a vital final obstacle that must be overcome for the effective translation of the technology from the laboratory to the market.

To connect the “last mile” between technical discovery and industrial application, future research and development must embrace a more engineering-focused and methodical methodology. At the technology integration level, investigating more stable biomimetic materials such as molecularly imprinted polymers (MIPs) as substitutes for antibodies, employing microfluidic chips for automated and standardized sample pretreatment to reduce matrix effects, and integrating artificial intelligence algorithms for analyzing multi-dimensional sensing data offer potential for collectively improving sensor robustness, throughput, and intelligence. At the ecosystem level, it is essential to advocate for the establishment of industry standards and certification protocols for biosensor-based rapid detection methods, while simultaneously dedicating efforts to the creation of affordable, integrated, and user-friendly portable devices for grassroots application, to facilitate the widespread adoption and sustainable incorporation of this technology into the global tea industry chain.

## 5. Summary and Prospect

This paper presents a thorough overview of the progress and prospective uses of biosensing technology for the detection of pesticide residues and heavy metals in tea. Conventional detection techniques, such as chromatography and mass spectrometry, provide exceptional accuracy in tackling the quality and safety issues related to pesticides, including pyrethroids, organophosphates, and neonicotinoids in tea, as well as heavy metals like lead, cadmium, and mercury. Nonetheless, these systems are constrained by their intricate operation, prolonged detection cycles, substantial instrument costs, and difficulties in on-site implementation. Biosensing technology has notable benefits, such as increased sensitivity, specificity, fast response, and operational simplicity, making it an essential instrument for the prompt screening and on-site identification of trace contaminants in complex tea matrices.

### 5.1. Research Summary

Biosensors exhibit diverse technological merits tailored to specific tea contaminant detection needs. Fluorescent biosensors, leveraging ratiometric designs and “signal-on/off” strategies, achieve nM-level sensitivity for heavy metals (e.g., Cd^2+^, Hg^2+^) and organophosphorus pesticides, with aptamer/MOF integration enhancing specificity. Similarly, surface-enhanced Raman spectroscopy (SERS) capitalizes on molecular vibration fingerprints for pesticide identification (e.g., imidacloprid), where engineered Au/Ag nanostructures (e.g., hedgehog-like arrays) amplify signals by 10^7^–10^8^-fold. Colorimetric sensors exploit LSPR-driven visual aggregation of Au/Ag nanoparticles for in situ heavy metal analysis, while core–shell designs (e.g., Fe_3_O_4_@MnO_2_) suppress matrix interference.

Advanced platforms enable real-time and ultrasensitive monitoring. SPR biosensors, combining label-free molecular interaction tracking with ion imprinting, detect heavy metals (Ni^2+^, Cd^2+^) at pM concentrations. Electrochemical sensors, including impedance-based and aptamer-nanolabel systems, offer portable, cost-effective solutions with fM-level pesticide detection (e.g., imidacloprid). Additionally, piezoelectric biosensors (e.g., QCM, EMPAS) quantify biomolecules and pesticide residues via mass-frequency correlations, enabling label-free, real-time analysis.

These technologies—summarized in Table 4—differ in sensitivity, specificity, and operational complexity, guiding their optimal deployment across laboratory, field, and industrial settings. Innovations in nanostructures (e.g., core–shell architectures) and signal amplification (e.g., SERS substrates) continue to bridge gaps between laboratory precision and on-site applicability.

### 5.2. Existing Challenges

Although considerable advancements in biosensor technology, the actual implementation of tea contaminant detection continues to encounter numerous crucial obstacles. A major constraint arises from the intricate matrix interference induced by tea polyphenols, alkaloids, and colors, which often nonspecifically adsorb onto sensing surfaces or impede biorecognition elements (e.g., enzymes, aptamers). This interaction undermines detection accuracy by distorting signals and diminishing the signal-to-noise ratio. Moreover, maintaining stability and repeatability is challenging owing to biomolecule breakdown during storage and operation, irregular nanomaterial dispersion, and variability in sensor manufacturing between batches, all of which hinder standardization efforts.

A significant limitation is the restricted multi-component detection capabilities of existing biosensors. Most are designed to target only a single or few contaminants, failing to meet the real-world demand for simultaneous screening of diverse pesticide residues and heavy metals in tea. Despite progress in high-throughput systems, scalable solutions are still inadequately developed. Moreover, the trade-offs between cost and mobility impede widespread adoption. Although portable devices are available, high-sensitivity technologies like as SPR and SERS continue to depend on cumbersome apparatus and expert handling. The absence of cost-effective, accessible point-of-care testing (POCT) alternatives limits availability for small-scale tea farmers and rural establishments.

Overcoming these issues necessitates advancements in interference-resistant biorecognition interfaces, reliable nanomaterial manufacturing, and multiplex sensor architectures. Future initiatives must focus the enhancement of portable, cost-effective detecting technologies to connect laboratory research with field implementation. Surmounting these obstacles will be crucial for guaranteeing food safety oversight across all tiers of tea manufacturing.

### 5.3. Future Outlook

Future biosensor development must emphasize unique recognition elements and signal amplification methodologies to surmount existing limits. Biomimetic materials such as molecularly imprinted polymers and peptide aptamers provide durable, economical substitutes for traditional antibodies, whilst advanced nanomaterials (MOFs, COFs, MXenes) may improve sensitivity and anti-interference characteristics via optimal signal transduction. Integrating these with multimodal detection methods (e.g., SERS-electrochemical, fluorescence-colorimetric hybridization) and microfluidic systems might automate sample preparation, enhance accuracy via cross-validation, and reduce matrix effects. These developments are essential for attaining dependable, high-throughput contaminant screening.

The integration of biosensing with digital technologies—such as IoT, AI, and big data analytics—will provide sophisticated, real-time monitoring systems across tea supply chains. The use of intelligent sensing nodes including remote data transmission and predictive functionalities has the potential to transform quality control from agriculture to consumption. A simultaneous emphasis on sustainability and standardization is crucial; the development of eco-friendly materials (e.g., biodegradable nanosensors) and standardized performance assessment methodologies will guarantee repeatability, commercial feasibility, and environmental compatibility.

Interdisciplinary cooperation across materials science, biotechnology, and information technology will be crucial in tackling ongoing difficulties such as sensitivity-stability trade-offs, multiplex detection, and field adaptation. Integrating these advances will enable the sector to evolve towards intelligent, cost-effective, and scalable monitoring systems, therefore ensuring the sustainability of the global tea industry and protecting public health.

## Figures and Tables

**Table 1:** Systematic classification of pesticide residues in tea with chemical types, target modes, detection rates and environmental behavior.

Category	Specific Name	Positive Rate	Mechanism of Action/Characteristics	Usage Regulation	Pollution Pathway	Reference
Pyrethroids	Bifenthrin	26.4%	Interferes with insect nerve conduction	Permitted, must comply with GB 2763-2021 standard	Direct pollution (foliar spray)	[23]
	Fenpropathrin	21.7%	Interferes with insect nerve conduction	Permitted, must comply with GB 2763-2021 standard	Direct pollution (foliar spray)	[24]
	Fenvalerate	-	Interferes with insect nerve conduction	Banned pesticide	Direct pollution (foliar spray)	[20]
Organophosphorus and Carbamates	Omethoate	-	Inhibits acetylcholinesterase activity in the insect nervous system	Restricted use	Exhibits both direct and indirect pollution characteristics	[25]
	Carbofuran	-	Inhibits acetylcholinesterase activity and interferes with nerve signal transmission in the insect nervous system	Restricted use	Exhibits both direct and indirect pollution characteristics	[26]
Neonicotinoids and Heterocyclics	Imidacloprid	15.1%	Interferes with the function of nicotinic acetylcholine receptors in the insect nervous system	Permitted, must comply with GB 2763-2021 standard	Exhibits both direct and indirect pollution characteristics	[27]
	Dinotefuran	6.67%	Interferes with insect nervous system function	Permitted, must comply with GB 23200.51-2016 standard	Exhibits both direct and indirect pollution characteristics	[28,29]
Fungicides and Herbicides	Carbendazim	15.1%	Interferes with fungal cell division and metabolic processes	Permitted, must comply with GB 23200.113-2021 standard	Exhibits both direct and indirect pollution characteristics	[30,31]
	Glyphosate	9.4%	Inhibits specific enzyme activity in plants, blocking the biosynthesis of aromatic amino acids	Restricted pesticide	Indirect pollution (migration via soil, water)	[32]
Organochlorines	Dicofol	-	Interferes with nervous system function of mites, ultimately leading to death	Banned pesticide	Indirect pollution (migration via soil, water)	[33]
	DDT	-	Interferes with insect nervous system function	Banned pesticide	Indirect pollution (migration via soil, water)	[34]

**Table 2:** Classification of heavy metals in tea: chemical nature, toxicity level, health impact, and regulatory limits.

Classification Dimension	Specific Type	Metal Name	Key Characteristics
Chemical Property and Toxicity Grade	Highly toxic heavy metals	Lead (Pb)	Exposure results in neurotoxicity, anemia, nephrotoxicity, hypertension, and developmental toxicity
		Cadmium (Cd)	Renal accumulation include irreversible renal damage and bone disease, notably osteoporosis accompanied by severe pain
		Mercury (Hg)	Tremors and cognitive decline, and is a developmental toxicant that causes severe defects upon fetal exposure
		Chromium (Cr^6+^)	Exposure via inhalation is carcinogenic, oral exposure leads to systemic organ damage, and dermal contact causes skin ulcers
		Metalloid Arsenic (As)	Human carcinogen and a chronic health hazard, associated with skin lesions, neuropathy, and multi-organ toxicity
	Moderately toxic heavy metals	Copper (Cu)	A toxicant capable of inducing gastrointestinal distress, abdominal pain, and, at high doses, hemolytic anemia and organ toxicity
		Zinc (Zn)	A disruptor of essential metal metabolism, leading to anemia and impaired immune function
		Nickel (Ni)	Contact dermatitis and an elevated cancer risk from chronic inhalation
Environmental Migration Characteristics	High mobility heavy metals	Cadmium (Cd)	High mobility and ion exchange enable its efficient uptake by tea roots from soil
		Zinc (Zn)	Ahigh soil-to-tea plant transfer factor due to efficient root uptake
	Low mobility heavy metals	Lead (Pb)	Immobile in soil, contamination of tea via adhesion and surface deposition
		Chromium (Cr)	Low solubility and mobility in soil limit root uptake, posing potential risks through alternative pathways
Pollution Source Pathway	Primary pollution source	Lead (Pb), etc.	Originating from geological weathering and soil parent materials
	Secondary pollution source	Cadmium (Cd), Mercury (Hg), Chromium (Cr^6+^), etc.	An anthropogenic pollutant primarily derived from industrial and agricultural activities

**Table 3:** Comparison Table of Soil Heavy Metal Limits in Chinese Tea Regions.

Pollutant Name	Tea Production Area Soil Environmental Limit	Screening Value for Soil Risk in Agricultural Land
Cd	pH ≤ 6.5:0.30; pH > 6.5:0.40	pH ≤ 5.5:0.3; 5.5 < pH ≤ 6.5:0.3
Pb	pH ≤ 6.5:250; pH > 6.5:300	pH ≤ 5.5:70; 5.5 < pH ≤ 6.5:90
Hg	pH ≤ 6.5:0.30; pH > 6.5:0.50	pH ≤ 5.5:1.3; 5.5 < pH ≤ 6.5:1.8
As	pH ≤ 6.5:40; pH > 6.5:30	pH ≤ 5.5:40; 5.5 < pH ≤ 6.5:49
Cr	pH ≤ 6.5:150; pH > 6.5:200	pH ≤ 5.5:150; 5.5 < pH ≤ 6.5:150

**Table 4:** Evaluation of performance and cost of various biosensors for target detection in tea and associated samples.

Detection Method	Linear Range	LOD (μg/L)	Recognition Material	Detection Time (T)/Reproducibility (RSD or CV)/Stability (S)	Real Samples	Equipment Cost	Material Cost	Reference
Fluorescence	0.01–10 μg/L (Cd^2+^)	0.001	Cd^2+^ aptamer	T = 25 min; RSD ≤ 5.0%; tea spiked recovery 88.2–102.5%	Tea, rice	$8000–15,000	$50–100	[75]
SERS	0.1–100 μg/L (imidacloprid)	0.05	Imidacloprid antibody	T = 5 min; RSD = 6.2%; tea spiked recovery 94.3–101.5%	Tea, fruits/vegetables	$150,000–180,000	$50–200	[76]
Colorimetric assay	0.01–50 μg/L (Hg^2+^)	0.005	Horseradish peroxidase	T = 20 min; RSD = 3.8%; tea spiked recovery 95.2–103.7%	Tea, environmental water	$5000–10,000	$30–80	[77]
SPR	0.01–1000 μg/L (Cd^2+^)	1.25	Ion-imprinted polymer	T = 15 min; RSD = 5.3%; tea/soil spiked recovery 92.6–100.8%	Tea, soil	$200,000–250,000	$500–800	[78]
Electrochemical Biosensors	0.01–100 μg/L (acetamiprid)	0.005	Acetamiprid aptamer	T = 20 min; RSD = 4.5%; tea/fruits spiked recovery 89.4–102.1%	Tea, fruits/vegetables	$6000–10,000	$30–80	[79]
Piezoelectric Biosensors	0.1–1000 μg/L (carbaryl)	0.1	Carbaryl antibody	T = 30 min; RSD = 7.5%; tea/pesticide samples recovery 91.3–101.2%	Tea, pesticide samples	$100,000–150,000	$150–300	[80]

**Table 5:** Performance Comparison and Application Scenarios of Biosensors for Tea Contaminant Detection.

Sensing Technology	Key Advantages	Key Limitations/Challenges	Typical Targets	Suitable Scenarios
Fluorescence	High sensitivity (nM-pM), rapid response, multiplexing potential	Photobleaching, matrix background interference, biotoxicity of some nanomaterials	Cd^2+^, Hg^2+^, Organophosphates	Laboratory precision analysis, High-throughput screening
SERS	“Fingerprint” specificity, single-molecule level sensitivity	Substrate reproducibility and cost, difficulty in signal quantification	Imidacloprid, other pesticides	Laboratory trace identification and quantification
Colorimetric	Visual readout, simple operation, very low cost	Limited sensitivity and accuracy, prone to sample color interference	Hg^2+^, Cu^2+^, Organophosphates	On-site rapid preliminary screening, Grassroots testing
SPR	Label-free, real-time kinetic monitoring, high precision	Expensive equipment, sensitive to nonspecific adsorption, poor portability	Cd^2+^, Ni^2+^, small molecule pesticides	Laboratory molecular interaction studies, High-precision detection
Electrochemical	High portability, excellent sensitivity, low cost, easy miniaturization	Electrode modification stability, interference from complex matrices (e.g., tea polyphenols)	Acetamiprid, Neonicotinoids	On-site rapid, portable quantitative detection
Piezoelectric	Label-free, real-time, highly sensitive to mass change	Long baseline stabilization time, high instrument cost, vulnerable to environmental vibration	Carbaryl, other pesticides	Laboratory real-time mass change monitoring

## Data Availability

The data presented in this study are available on request from the corresponding author. The data are not publicly available due to privacy.
